# Neuropsychological functioning in late-life depression

**DOI:** 10.3389/fpsyg.2013.00381

**Published:** 2013-06-27

**Authors:** Gro Strømnes Dybedal, Lars Tanum, Kjetil Sundet, Torfinn Lødøen Gaarden, Tor Magne Bjølseth

**Affiliations:** ^1^Department of Geriatric Psychiatry, Diakonhjemmet HospitalOslo, Norway; ^2^Department of Research and Development in Mental Health, Akershus University HospitalLørenskog, Norway; ^3^Department of Psychology, University of OsloOslo, Norway

**Keywords:** late-life depression, executive function, memory, information processing speed, neuropsychological

## Abstract

**Background:** The literature describing neurocognitive function in patients with late-life depression (LLD) show inconsistent findings in regard to incidence and main deficits. Reduced information processing speed is in some studies found to explain deficits in higher order cognitive function, while other studies report specific deficits in memory and executive function. Our aim was to determine the characteristics of neuropsychological functioning in non-demented LLD patients.

**Methods:** A comprehensive neuropsychological battery was administered to a group of hospitalized LLD patients and healthy control (HC) subjects. Thirty-nine patients without dementia, 60 years or older meeting DSM-IV criteria for current episode of major depression, and 18 non-depressed control subjects were included. The patient group was characterized by having a long lasting current depressive episode of late-onset depression and by being non-responders to treatment with antidepressants. Neurocognitive scores were calculated for the domains of information processing speed, verbal memory, visuospatial memory, executive function, and language. Number of impairments (performance below the 10th percentile of the control group per domain) for each participant was calculated.

**Results:** Nearly half of the patients had a clinically significant cognitive impairment in at least one neurocognitive domain. Relative to HC subjects, LLD patients performed significantly poorer in the domains of information processing speed and executive function. Executive abilities were most frequently impaired in the patient group (39% of the patients). Even when controlling for differences in processing speed, patients showed more executive deficits than controls.

**Conclusions:** Controlling for processing speed, patients still showed impaired executive function compared to HCs. Reduced executive function thus appears to be the core neurocognitive deficit in LLD. Executive function seems to be an umbrella concept for several connected but distinct cognitive functions. Further studies of neuropsychological functioning in LLD patients are needed to characterize more specific what kinds of executive impairments patients have. Additional studies of remitted LLD patients are needed to separate episode-related and persistent impairments.

## Neuropsychological functioning in late-life depression

Late-life depression (LLD) refers to the presence of a significant clinical depression in individuals over 60 years of age and is typically defined independently of age at onset (O'Hara et al., 2006). When studying neurocognition in major depression, elderly patients should be identified as a separate group because their age makes them psychobiologically different from younger individuals (Bryan and Luszcz, 2000). Neurocognition in elderly non-demented patients with major depression is characterized by considerable heterogeneity. According to Butters et al. (2004) and Bhalla et al. (2009) about 40–60% of non-demented patients with late life depression can be classified as cognitively impaired after thorough neuropsychological assessment. However, a considerable number of LLD patients show no significant sign of cognitive impairment. Cognitive deficits tend to persist in the remitted state (Bhalla et al., 2006; Köhler et al., 2010), although in a subgroup of depressed elderly patients with cognitive dysfunction, cognition may improve somewhat in remitters (Butters et al., 2000).

Persistent cognitive deficits after remission in patients with LLD may be related to neurobiological changes, including brain atrophy and an increased prevalence of white matter hyperintensities (Wilkins et al., 2009). There is also increasing evidence for a link between LLD and development of dementia, included Alzheimer disease (Steffens et al., 2006). Cognitive deficits in LLD have been associated with increased rates of relapse of depression, disability and poorer response to antidepressant treatment (O'Hara et al., 2006).

The characteristic cognitive profile of this group of patients and possible mechanisms causing impairment, are topics of discussion in the literature. In a recent review, Herrmann et al. (2007) reports that a large proportion of LLD patients suffer from reduced executive function, processing speed, episodic memory, and semantic memory. Visuospatial ability, attention and inhibition, working memory and expressive language have also been observed to be impaired in patients with LLD (O'Hara et al., 2006). Executive dysfunction has systematically been listed as the most pronounced deficit among LLD patients (Herrmann et al., 2007). These findings support the theory put forth by Alexopoulos et al. (2000) stating that frontostriatal dysfunction contributes both to the development of LLD and to executive dysfunction. Differences between LLD patients with early and late onset are frequently not found (Butters et al., 2004; Sheline et al., 2006).

Although frequently referred to as a unitary function (Alexopoulos et al., 2000; Butters et al., 2004), executive function seems to be an umbrella concept for several connected but distinct higher cognitive functions (Miyake et al., 2000a; Hull et al., 2008). In accordance with advices to clinicians from Miyake et al. (2000b), we have made a composite measure of executive function to be used in our analysis in order to reduce some of the weaknesses in reliability and validity of individual measures. We have chosen to classify verbal fluency tests as a language domain. Verbal fluency tests are often listed within the executive function domain, but have been reported as rather insensitive measures of executive function (Henry and Crawford, 2005; Rodriguez-Aranda and Sundet, 2006), and are thus treated separately in our study.

Butters et al. (2004) maintain that reduced information processing is responsible for more specific neuropsychological deficits in LLD, including executive dysfunction. Sheline et al. (2006) also found that slowed processing speed appeared to be the core deficit in LLD. Nebes et al. (2000) noted that reductions in processing resources (speed and working memory) appeared to persist following remission of depression and that these kind of deficits may be trait markers for LLD. Other cognitive deficits could be explained by reductions in processing resources. Sexton et al. (2012) concludes that impairments in executive function or processing speed were sufficient to explain differences in episodic memory and language skills in their group of (remitted) LLD patients. They also found that executive deficits could not be fully explained by impairments in processing speed.

Our aims are (1) to analyse and describe neuropsychological function in LLD patients, and to compare this group with healthy controls (HCs). We will focus on the domains of information processing speed, memory, verbal fluency, and executive function. Our hypothesis is that depressed patients are characterized by cognitive deficits in all of these domains, but have the most pronounced deficits in information processing speed and executive function. (2) We want to examine the incidence of cognitive impairment in LLD patients by calculating the number of impaired cases in the patient group compared to the control group. (3) We also want to investigate if a general deficit, like information processing speed slowing, is a main deficit that can explain deficits in other more specific cognitive domains, including memory and executive function.

## Materials and methods

### Participants

Norwegian speaking Caucasian inpatients meeting the DSM-IV criteria of major depression (single episode, recurring depression or bipolar disease) during the period 01.09.09 to 20.12.12 were asked to join the study, unless they met the exclusion criteria. The study was approved by the Regional Committee for Research Ethics (REK) in Norway which included an approval of the use of ECT as a first-line medical treatment in selected cases. In this paper we present baseline data from testing of a group of inpatients that some days after testing was given ECT. They were also tested after ECT-series and after an additional 3 months. Inclusion criteria were age 60–85 years old, being hospitalized in the Department of Geriatric Psychiatry (DGP), Diakonhjemmet Hospital, and providing a written consent to participate in baseline testing and later randomization to bifrontal or unilateral electrode placement in electroconvulsive therapy (ECT). Patients were excluded if (1) having a Mini Mental State Examination (MMSE) Score (Folstein et al., 1975) of less than 24 (maximum 30 points), as this could be a strong indication of dementia, (2) a diagnosis of dementia or other neurodegenerative disorder within the follow-up period of 5 months. Patients with clear signs of cognitive impairment at baseline, were followed especially closely and underwent an interdisciplinary dementia assessment, (3) other diagnosis of neurological disorder; e.g., head injury, stroke or Mb. Parkinson, (4) current or earlier substance abuse, (5) rapid cycling bipolar disorder, (6) schizophrenia or schizoaffective disorder. Our criteria for participation in the study allowed patients with mild cognitive impairment (either amnestic or other type) to be included in the study, because older adults with depression often present with signs and symptoms indicative of cognitive impairment (Wilkins et al., 2009). (7) Since the patients were given ECT-treatment after baseline-testing, there were some exclusion criteria related to this kind of treatment, e.g., that ECT has not had any effect in an earlier depressive episode or ECT treatment during the last 6 months. In order to relate cognitive function of LLD patients to an age-matched group without a history of psychiatric illness, we recruited 20 elderly patients from a recreational center in the community. Eighteen of these patients had a MMSE score of 24 or better at baseline and were included as controls.

### Clinical assessment

Clinical assessment at admission was carried out by a trained psychiatrist (TMB or TLG) who also scored Hamilton Depression Scale (17-items) (HAM-D 17, Hamilton, 1960). TMB also assessed all the patients with MINI International Neuropsychiatric Interview; specifically the MINI-Plus (Sheehan et al., 1998) before inclusion. He had participated in a structured training program for MINI-Plus. Patients with a higher score than 17 points on HAM-D 17 and diagnosis of major depression according to MINI could be included. Age at onset, number of previous episodes, other disease parameters and somatic morbidity were determined by the psychiatrist (TMB or TLG) from the clinical interview. A thorough examination of medical records and scoring of Cumulative Illness Rating Scale for Geriatric Patients (CIRS-G) (Miller et al., 1992) was also performed. For our purposes, the psychiatric item of the CIRS-G was excluded. The psychiatrist (TMB) also administered HAM-D 17 and MMSE to controls, evaluated their somatic morbidity with CIRS-G and ascertained that they did not have a history of any major psychiatric illness.

### Neurocognitive assessment

Neurocognitive assessment of the patients and controls were carried out by the clinical neuropsychologist (GSD) or a nurse. The nurse had been trained by GSD in administering the test battery. Another trained nurse and an occupational therapist had received training by GSD and they assisted with testing of the HCs. The test battery was chosen with the main rationale of giving measurements of memory and executive function as a background for assessing cognitive adverse effects of ECT. Wanting patients to be motivated to go through testing three times within a period of about 5 months, we composed a test battery that was comprehensive, but not too strenuous, consisting of subtests from standard test batteries validated for Norwegian use. Learning and memory was measured using the official Norwegian research version of the Hopkins verbal learning test–revised (HVLT-R, Brandt and Benedict, 2001), and the Brief Visuospatial Memory test–R (BVMT-R, Benedict, 1997). Information processing speed was assessed by the Trail Making test part A (Reitan and Wolfson, 1993) and D-KEFS Color Word Interference test part 1 and 2 (CWIT, Delis et al., 2005). Aspects of executive function were assessed by D-KEFS Tower Test (Delis et al., 2005), the CWIT part 3 and 4 (Delis et al., 2005) and the Trail Making test part B (Reitan and Wolfson, 1993). The CWIT part 3 is based on the Stroop (1935) procedure. Verbal fluency was assessed with the letter fluency from the D-KEFS battery (Delis et al., 2005) and the animal naming test (Borowski et al., 1967). The subtest Vocabulary from the Wechsler Abbreviated Scale of intelligence (WASI) (Wechsler, 2007) was used as an estimate of premorbid intelligence. Global cognitive function was assessed by a revised version of the MMSE by Engedal et al. (1988). Patients who did not manage the serial subtraction by sevens in MMSE were not tested with backward spelling, which was assumed to be a more easy measure of attention (Ganguli et al., 1990). A self-constructed unpublished “Media questionnaire” consisting of 20 questions was intended for repeated assessments studying retrograde amnesia for information presented in the news prior to treatment (2003–2008). The questionnaire served also as a measure of general public knowledge among patients and controls. An adapted version of the Autobiographical Memory Interview–Short Form (AMI-SF, McElhiney et al., 1997) measuring personal semantic memories was administered. It is designed for repeated use after ECT-treatments and measures retrograde amnesia for autobiographical memories. It is commonly used in ECT-studies, but has lately been criticized because of weak separation between normal forgetting over time and retrograde amnesia and lack of validiation studies (Semkovska and McLoughlin, 2013). We adapted AMI-SF for older subjects before the start of the study. After data from the control subjects were collected, we deleted five items that could not be answered consistently 2 months after baseline by a majority of healthy elderly. These items seemed to threaten the validity of AMI-SF as a measure of retrograde amnesia. Maximum score in our adapted version is 30 compared to 60 in the original McElhiney et al. (1997) version.

There were little missing data, except for The Trail Making test part B that was too difficult for 8 patients who gave up before the task was completed. Results are also missing for two controls on this test. One patient and two controls did not complete the CWIT part 1 and 2, three patients and two controls did not complete part 3 and six patients and two controls did not complete part 4. There are missing scores on the Tower Test for one patient and one control. In addition, there is one missing score for controls on the Trail Making Test part A and one missing score for patients on the Animal Naming Test and the Letter Fluency Test.

Our test battery was organized into 5 domains based mainly on the current knowledge about what each test measures (Strauss et al., 2006; Lezak et al., 2012). Raw scores were transformed into *z*-scores, using the means and standard deviations of the elderly control sample. The variance in the control group's scores was checked and we found no major threat to validity of the transformed *z*-scores. Missing scores were replaced with the average score for the group (patient or control). The *z*-scores were then averaged within each neuropsychological area to produce domain scores. The domain Information processing speed consists of the CWIT part 1 and 2 and the Trail Making test part A. The domain of Verbal memory consists of 3 scores from the HVLT-R; total learning, delayed recall and discrimination (recognition). The domain of visuospatial memory consists of three scores from the BVMT-R; total learning, delayed recall and discrimination. The domain of language consists of scores from two fluency tests; the animal learning test and the letter fluency. The executive domain consists of the Trail Making test part B, the D-KEFS Tower test and the CWIT part 3 (time + errors divided by two). The CWIT part 3 time and the CWIT part 3 errors were highly correlated (*r* = 0.486, *p* < 0.001), supporting that the measures are related, although they measure different aspects of performance. We chose to exclude part 4 of the CWIT from our analyses. The CWIT 4 was designed to be the most difficult part of the test (Delis et al., 2005). But several studies question the validity of the CWIT 4 as an equally or more sensitive test of executive impairment than the CWIT 3 in clinical groups (Lippa and Davis, 2010; Savla et al., 2011). In addition, several patients in our study did not comply with the CWIT 4 at the 3 months follow-up, although they did at baseline. Including the CWIT 4 within the baseline domain score, would leave us with an executive function score that could not be used at the follow-up. Another reason to exclude the CWIT 4 was more missing scores at baseline. The CWIT 3 time and the CWIT 4 time scores were highly correlated (*r* = 0.464, *p* = 0.001), supporting the expectation (Delis et al., 2005) that they partly measure the same executive function (inhibition). Chronbach's alpha and mean inter item correlation for domain scores were: Information processing domain (α = 0.72/*r* = 0.48), Verbal memory (α = 0.85/*r* = 0.65), Visuospatial memory (α = 0.86/*r* = 0.70), Executive function (α = 0.73/*r* = 0.51), and Language (α = 0.42/*r* = 0.27). The number of domains (0–5) in which each subject was impaired (defined as performing below the 10th percentile of the control group) was calculated for both groups.

### Statistical analysis

The Statistical Package for the Social Sciences (SPSS Inc., Chicago, IL; version 20.0) was used. Means and standard deviations are reported for continuous variables and percentages for categorical variables. Clinical data were statistically analyzed using conventional descriptive methods and *t*-tests. Statistical significance was determined using the 0.05 level and 2-tailed tests of significance. Dichotomous variables were analyzed with crosstabs with corresponding *post hoc* analyses performed using chi-squares. In situations where the expected cell frequencies were <5, Fisher's exact test was used. Correlation analyses (Spearman) explored whether neurocognition was associated with demographic variables or clinical variables. One-way ANCOVAs with age as a covariate were used to compare the groups on continuous variables. Multivariate tests were not used on neurocognitive raw scores because of missing data, especially on the Trail Making test. The relative impact on neurocognition by diagnosis of LLD was investigated by a one way MANCOVA with all 5 neurocognitive domain scores entered as dependent variables. Age was entered as a covariate. Then, a MANCOVA with information processing speed and age as covariates was conducted, investigating whether variability in neurocognitive function would then be explained by reduced processing speed. We followed-up significant findings in the MANCOVAs by single-test analysis of covariance. Effect sizes are reported as partial eta squared. Bonferroni corrections were performed by dividing the p-value by number of tests/domains. Lastly, a comparison of number of cognitive impairments in depressed patients and controls was done with a Mann–Whitney *U*-test.

## Results

### Clinical characteristics of the patient group

Demographic and clinical characteristics of the LLD and HC groups are presented in Table 1. There were no statistically significant group differences between the LLD and HC participants regarding age, education, gender, estimated premorbid IQ (Vocabulary, WASI), global cognitive function (MMSE), total medical disease burden (CIRS-G) or vascular burden (CIRS-G heart and vascular score). There was a 3 year age difference between groups (*p* = 0.087) which may represent a possible confounder for neurocognitive performance. Group comparisons were therefore performed with age as covariate.

**Table 1:** **Characteristics of the study participants**.

**Characteristic**	**Patients with LLD (*n* = **39**)**	**Controls (*n* = **18**)**	**Test statistics**	
Age, mean (*SD*), *y*	74.5 (7.0)	77.7 (4.7)	*t*_55_ = 1.7	*p* = 0.087
Women, No. (%)	22 (56.4)	12 (66.7)	χ^2^_1_ = 0.5	*p* = 0.463
Education, mean (*SD*), *y*	13.2 (2.9)	13.1 (3.1)	*t*_55_ = 0.1	*p* = 0.936
Vocabulary (WASI) *t*-score, mean (*SD*)	56.3 (7.8)	57.7 (8.2)	*t*_55_ = 0.6	*p* = 0.541
MMSE score mean (*SD*)	27.6 (1.9)	28.6 (1.5)	*t*_55_ = 1.9	*p* = 0.064
Media questionnaire, mean (*SD*)^a^	28.5 (5.5)	33.1 (5.6)	*t*_55_ = 3.0	*p* = 0.005
AMI-SF mean (*SD*)^b^	28.7 (2.1)	28.9 (2.3)	*t*_55_ = 0.3	*p* = 0.781
HAM-D 17 mean (*SD*)	26.5 (4.2)	2.3 (2.5)	*t*_54_ = 22.6	*p* < 0.001
CIRS-G total score, mean (*SD*)	5.5 (3.4)	5.3 (2.4)	*t*_55_ = 0.1	*p* = 0.887
CIRS-G heart and vascular score, mean (*SD*)	1.1 (1.1)	1.3 (1.3)	*t*_55_ = 0.4	*p* = 0.657
Severe depression according to HDRS-17 (%)	26 (66.7)			
Single episode, No. (%)	8 (20.5)			
Unipolar depression, No. (%)	25 (64.1)			
Bipolar II, No. (%)	6 (15.4)			
Psychosis, No. (%)	11 (28.2)			
Age of first depression episode, mean (*SD*), *y*	55.5 (18.7)			
Duration of current episode (weeks)	42.6 (37.4)			
Number of adequate trials with antidepressant, this episode (*SD*)	1.5 (1.0)			
Somatic syndrome, No. (%)	37 (94.9)			
Number of major depressive episodes lifetime, mean (*SD*)	2.5 (3.2)			
Number of previous hospitalizations in a psychiatric ward (*SD*)	1.3 (1.8)			

Abbreviations: AMI-SF, Autobiographical Memory Interview–short form; CIRS-G, Cumulative Illness Rating Scale–Geriatrics; HAM-D 17, Hamilton Depression Rating Scale–17 items; LLD, late-life depression; MMSE, Mini Mental State Examination; WASI, Wechsler Abbreviated Scale of Intelligence.

a Self-constructed, unpublished. Maximum score is 40.

b Modified for use in this study. Maximum score is 30.

The majority of patients had a severe unipolar depression without psychosis, but with somatic syndrome. The mean age of first depression episode was in the mid-fifties. As a group, the patients had previously rather few life-time depressive episodes and few previous hospitalizations, but reported a long mean duration of the current episode. 46.1% of the patients could be considered as medication-resistant defined as no clinical response to previous treatment with at least two different antidepressive agents given in adequate doses over an adequate period of time. An additional 38.5% had no clinical response to previous treatment with one antidepressive agent given in adequate doses over at least 4 weeks.

### Neurocognitive performance

Table 2 presents raw scores on neuropsychological tests. Using age as a covariate in the statistical analyses of group differences, Table 2 shows that the differences were reaching the level of nominal significance in six of the 15 single measures. One of the single measures (Trail Making test part B) remained statistically significant after Bonferroni corrections (setting the *p*-value at 0.003). A more detailed analysis of performance on the Tower test showed that the number of correct solved towers were significantly different between the groups [*F*_(1, 52)_ = 10.63, *p* = 0.002]. There were no significant differences in how effective patients and controls were in solving the tower tasks they eventually succeeded with (moves used on tasks solved / minimum moves needed to solve these tasks) [Mean efficacy of patients 1.2 (0.3) and controls 1.23 (0.2), *F*_(1, 49)_ = 1.5, *p* = 0.229].

**Table 2:** Results of ANCOVA tests comparing LLD patients' and Control subjects' raw score performance on individual neurocognitive tests^*^^a^.

**Test**	**Patients (*n* = **39**)**	**Controls (*n* = **18**)**	**ANCOVA^a^**		**η^2^_*p*_**
			***F***	***P***	
**INFORMATION PROCESSING DOMAIN**					
C-W Interference—color naming^b^	41.9 (10.5)	35.3 (6.3)	7.1	0.011	0.12
C-W Interference—word reading^b^	27.5 (6.1)	27.6 (5.6)	0.0	0.838	0.001
Trail Making test A	63.4 (21.6)	53.1 (17.9)	5.2	0.027	0.09
**VERBAL MEMORY**					
HVLT-R total learning	21.1 (4.7)	21.9 (5.7)	1.6	0.218	0.03
HVLT-R delayed recall	7.1 (2.7)	7.5 (2.4)	1.2	0.282	0.02
HVLT-R discrimination^c^	10.7 (1.3)	10.8 (1.3)	0.1	0.714	0.003
**VISUOSPATIAL MEMORY**					
BVMT-R total learning	12.5 (5.7)	14.4 (6.9)	2.9	0.097	0.05
BVMT-R delayed recall	4.8 (2.7)	5.4 (2.5)	1.9	0.180	0.03
BVMT-R discrimination^c^	4.8 (1.2)	5.2 (1.0)	2.1	0.151	0.04
**EXECUTIVE FUNCTION**					
Tower test^b^	12.7 (4.1)	15.9 (3.9)	8.1	0.006	0.14
Trail Making test B	191.5 (78.4)	139.4 (61.0)	10.0	0.003	0.19
C-W Interference—inhibition, time^b^	100.7 (33.3)	82.8 (22.9)	5.6	0.022	0.10
C-W Interference—inhibition errors^b^	3.4 (4.4)	0.9 (1.1)	6.6	0.013	0.12
**LANGUAGE**					
Animal naming test	17.3 (5.5)	18.7 (6.1)	2.8	0.103	0.05
Letter fluency (F, A, S)^b^	38.4 (12.3)	39.2 (11.4)	0.01	0.931	<0.001

Abbreviations: BVMT-R, Brief Visuospatial Memory Test–revised; C-W interference, Color Word Interference Test (variant of the Stroop test); HVLT-R, Hopkins verbal learning test–revised.

* Data are given as mean (SD) of conventional scoring method.

a Age is used as a covariate.

b D-KEFS, Delis Kaplan Executive Function System.

c Recognition; number correct minus false positives.

Table 3 presents correlations between the raw scores and demographic variables for the patient group. There were several significant correlations between age and neurocognitive measures, confirming the benefit of using age as a covariate in further analysis. Correlations between cognitive measures and depression score show small to medium effects. Sex and education levels have modest effects on some tests, while somatic morbidity shows very small effects.

**Table 3:** **Pearson correlations between demographical variables and the patients' neuropsychological raw scores^a^**.

**Neuropsychological test**	**Sex**	**Age**	**Education**	**CIRS-G^b^**	**Depression^c^**
HVLT-R total learning	0.29	−0.26	0.19	−0.07	−0.34^*^
HVLT-R delayed recall	0.08	−0.25	0.05	0.04	−0.26
HVLT-R discrimination	−0.07	−0.10	0.15	0.07	−0.27
BVMT-R total learning	0.39^*^	−0.36^*^	0.26	−0.02	−0.26
BVMT-R delayed recall	0.33^*^	−0.39^*^	0.35^*^	−0.04	−0.20
BVMT-R discrimination	−0.09	−0.28	0.10	−0.09	−0.21
Tower test^d^	0.22	−0.25	0.06	−0.05	−0.22
C-W Interference—color naming^d^	0.04	0.16	0.24	0.03	0.20
C-W Interference—word reading^d^	0.04	0.15	0.04	0.11	−0.21
C-W Interference—inhibition, time^d^	−0.001	0.30	−0.30	−0.18	0.10
C-W Interference—inhibition, errors^d^	−0.21	0.23	−0.17	−0.15	0.12
Animal naming test	0.12	−0.37^*^	−0.07	−0.08	−0.08
Letter fluency (F, A, S)^d^	0.28	0.22	0.33^*^	0.25	−0.01
Trail Making test part A	−0.11	0.29	−0.16	0.09	−0.09
Trail Making test part B	−0.20	0.45^**^	−0.24	0.16	0.11

Abbreviations: BVMT-R, Brief Visuospatial Memory Tes–revised; C-W Interference, Color–Word Interference test (variant of the Stroop test); CIRS-G, Cumulative Illness Rating Scale–Geriatrics; HVLT-R, Hopkins verbal learning test–revised.

a Results are given for patients with LLD (n = 39).

b Total score.

c HAM-D 17, Hamilton Depression Rating Scale–17 items.

d D-KEFS, Delis Kaplan Function System.

* P < 0.05;

** P ≤ 0.01.

Bold values indicate the correlation between HVLT total learning and depression is −0.34 and significant.

A one-way between-groups multivariate analysis of variance was performed to investigate group differences in cognitive function, by domains. The results are shown in Table 4. Five dependent variables were used; the domain scores Information processing speed, Verbal memory, Visuospatial memory, Executive function, and Language. The independent variable was group (patient vs. controls), age was covariate. There was a statistically significant difference between LLD patients and controls on the combined dependent variables [*F*_(5, 50)_ = 3.7, *p* = 0.006, η^2^_*p*_ = 0.27]. When analyzing domain scores separately, Information processing and Executive function reached statistical significance, using an alpha level of 0.05, with LLD patients performing poorer than controls. After Bonferroni corrections the executive function domain remains significant.

**Table 4:** **Results on MANCOVA tests comparing elderly control subjects' and LLD patients' *z*-score performance on cognitive domains^a^**.

**Domain**	**Mean (*SD*)**	**F_(1, 54)_**	***p***	**η^2^_p_**
Information Processing Speed^b^	−0.5 (1.0)	5.9	0.019	0.10
Verbal Memory^c^	−0.1 (0.9)	1.0	0.318	0.02
Visuospatial Memory^d^	−0.3 (0.9)	3.0	0.087	0.05
Executive Function^e^	−1.1 (1.2)	17.4	<0.001	0.24
Language^f^	−0.15 (0.8)	0.9	0.338	0.02

Abbreviations: LLD, late-life depression.

a Results are given for the patients with LLD (n = 39), age is used as a covariate.

b Trail Making test A, D-KEFS C-W Interference test color naming and word reading (average score).

c Three measures from HVLT-R (average score).

d Three measures from BVMT-R (average score).

e Trail Making test B, D-KEFS Tower test, and (C-W interference—inhibition, time + C-W interference, inhibition, errors)/2 (average score).

f Animal naming test and D-KEFS Letter Fluency (F,A,S).

A second one-way between-groups multivariate analysis of variance with Information processing speed and age as covariates was performed for the four domain scores Verbal memory, Visuospatial memory, Executive function, and Language. The difference between LLD patients and controls remained statistically significant on the combined dependent variables, *F*_(4, 50)_ = 2.9, *p* = 0.030, η^2^_*p*_ = 0.19, as well as the Executive function when conducted separately [*F*_(1, 53)_ = 10.4, *p* = 0.002]. After Bonferroni corrections the executive function domain remains significant.

**Figure 1:**
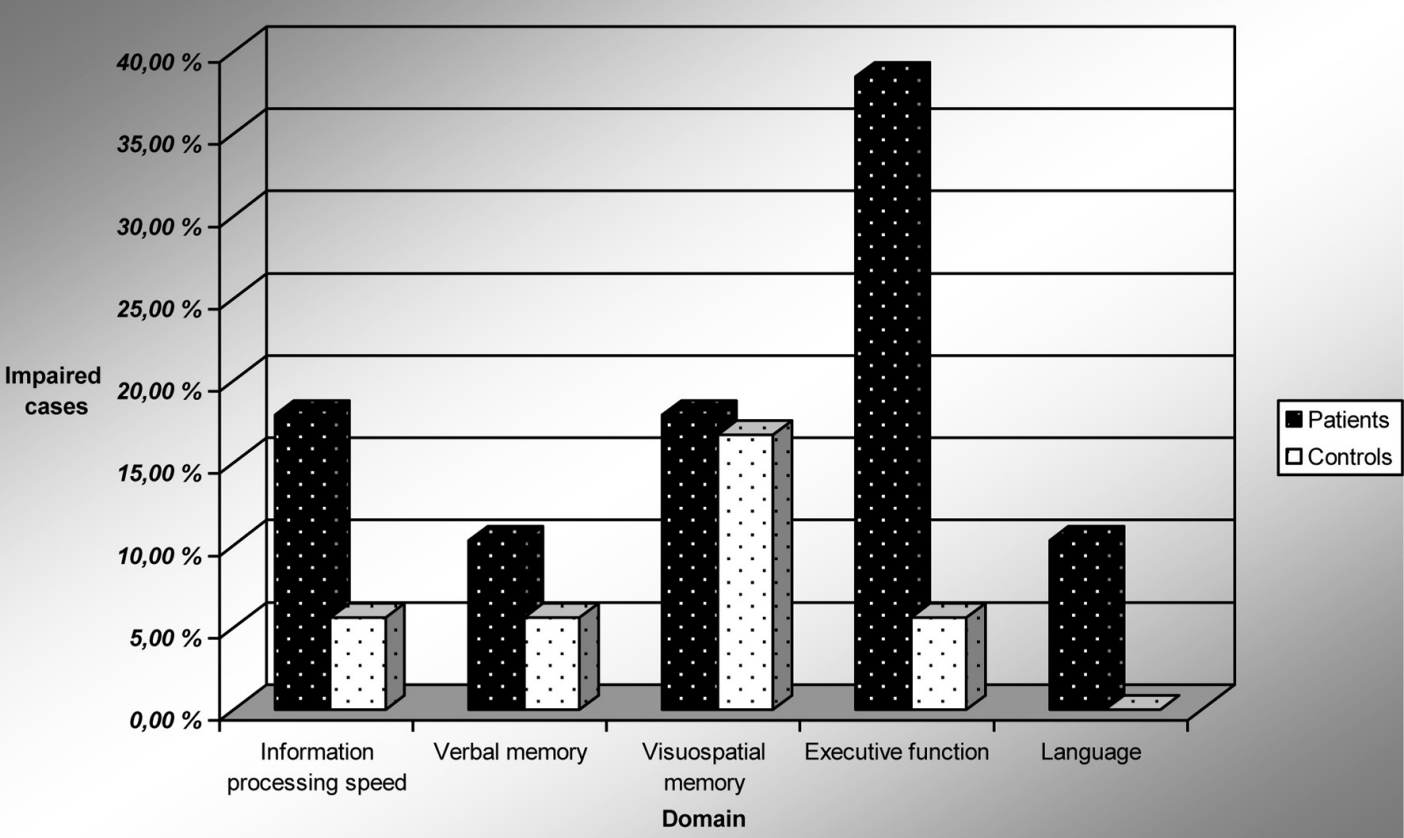
**Performance on cognitive domains by LLD patients and controls**. Impairment is defined as performance below the 10th percentile of the control subjects. Abbreviation: LLD, late life depression.

Investigating the number of impaired cases, we found that the depressed patients were impaired in more domains than control subjects (Mann–Whitney *U*-test, *p* = 0.05). Table 5 shows that in the patient group, close to 50% of the patients experienced a clinically significant impairment in at least one cognitive domain compared to approximately 20% of the controls. Figure 1 shows performance on cognitive domains by LLD patients and controls. More depressed patients were impaired in the executive domain (39 vs. 5.6% of controls, χ^2^ = 6.6, *p* = 0.010), but the differences did not reach significance in the other domains.

**Table 5:** **Percentage of Participants Exhibiting Cognitive deficits (defined as 10th percentil below comparison group) and mean number of deficits for patients with LLD and controls**.

**No. of deficits**	**Patients with LLD, % (n = 39)**	**Controls, % (n = 18)**
0	51.3	77.8
1	20.5	11.1
2	15.4	11.1
3	10.3	0
4	0	0
5	2.6	0
Total	100	100
Mean No. of deficits (*SD*)	1.0 (1.2)	0.3 (0.7)

Abbreviation: LLD, late-life depression.

## Discussion

Patients with late-life major depression were characterized by slower information processing speed and a markedly stronger degree of executive deficits than non-depressed HCs. When controlling for information processing speed, the difference in the domain of executive function remained significant. There were no statistically significant differences in verbal memory, visuospatial memory or language (verbal fluency) between groups. Our findings are consistent with others reporting main differences between such groups on tests of information processing speed and executive function (Herrmann et al., 2007).

Contrary to the findings reported by Butters et al. (2004), but in line with Sexton et al. (2012) and Sheline et al. (2006), we found that deficits in the executive function could not be fully explained by a general information processing speed deficit. Such differences between study reports may be partly explained by the composition of the test battery and how the composite neurocognitive measures are defined. Even more important is probably differences between patient groups. The main features of our inpatient group seem to be different from the outpatient group of Butters et al. (2004). They studied mainly outpatients not yet prescribed antidepressants at the time of testing and 50% of the patients experienced their first depressive episode. The LLD patients in our study were characterized by among other factors vegetative symptoms, non-responding to treatment with antidepressants, and a protracted course of current depressive episode. Non-remitters on antidepressants more often show executive deficits (Steffens and Potter, 2008; Snyder, 2013).

Consistent with the results of Meeter et al. (2011) the patients' scores on a test that assessed memory for news were lower than those of controls (Media Questionnaire). In accordance with Butters et al. (2004), but in contrast to O'Brien et al. (2004), we did not find significant differences between groups on tests of verbal fluency. We did not find any significant difference between the groups in the memory domain either, in contrast to what has been reported by others (Butters et al., 2004; Köhler et al., 2010; Mesholam-Gately et al., 2012). Our results underline that neurocognitive impairment in non-demented LLD patients does not necessarily include memory impairment. Given that one out of several common pathways linking LLD, mild cognitive impairment and at a later stage dementia, may be cerebrovascular disease and Alzheimer neuropathology (Butters et al., 2008), it is natural to expect memory impairment at least in subgroups of non-demented LLD patients. A likely explanation of different results within the memory domain, are differences in patient and control populations and in inclusion and exclusion criteria. Butters et al. (2004) state that they were “using minimal exclusionary criteria to maximize the heterogeneity among LLD patients, thus permitting evaluation of a range of potential risk factors for cognitive impairment” (p. 593). Our choice of exclusionary criteria appears to have been more comprehensive.

In our study, significantly more depressed patients were cognitively impaired than HC subjects. More depressed patients had executive deficits, but the differences did not reach statistical significance in the domains of verbal memory, visuospatial memory, information processing speed and language. In our study 51.3% of the LLD patients were unimpaired, vs. 77.8% of the controls. In comparison, Butters et al. (2004) found that 39% of their LLD patients and 67.5% of their controls were unimpaired. Common for the studies is that a substantial part of LLD patients have cognitive deficits.

The largest effect sizes when comparing LLD patients and controls were seen on the Tower test and the Trail Making test part B. These tests are speed-dependent. But in addition they require executive abilities like mental flexibility, planning and working memory. Several studies have found that LLD patients have lower scores on TMT B (e.g., Lockwood et al., 2002; O'Brien et al., 2004). Our findings are conservative because we choose to record lacking capability to carry through the test as a missing score and not maximum score.

Mood disorders, and in particular depressive disorders, have been believed to impact overall executive functioning. The Tower test is regarded as a sensitive test of this domain, but very few studies have actually examined the effects of mood disorders on Tower performance (Sullivan et al., 2009). As shown in our study, the patients were just as effective as controls (total moves/minimum moves) when working on Tower tasks that they solved, but they gave up more quickly and solved fewer items. There was also no difference between groups with regard to rule infractions while performing the test.

## Conclusions

Non-demented patients with LLD were significantly more cognitively impaired than HCs. Nearly 50% of patients showed clinically significant impairment (performance below the 10th percentile of the control group) in at least one of five neurocognitive domains. Approximately 40% of the patients showed deficits in the domain of executive function and nearly 20% in the domain of information processing speed, but in other cognitive domains they were not significantly more impaired than controls. When Information processing speed is accounted for, patients still show deficits in executive function. The Tower Test and the Trail Making Test part B seem to be especially sensitive tests for detecting cognitive deficits in LLD-patients. Future studies are needed to clarify the exact nature of the executive deficits and the degree of persistence of deficits after successful clinical treatment. Numerous studies have documented the weaker effect of antidepressants in the subgroup of LLD-patients with executive deficits (Story et al., 2008), but there is a lack of studies on the effect of ECT-treatment in this group. There are several clinical implications of our findings. LLD patients ought to be screened routinely for cognitive impairment at admission at a DGP. Results from screening tests and in particular from more detailed neuropsychological assessment can assist therapists in tailoring treatment and follow-up to the patients' individual needs. Realistic expectations and tailored treatment can hopefully make relapse of depression after remission less likely.

## Strengths and limitations

A strength of our study is the exclusion of patients with suspected dementia or other neurological diseases at admission. By following up the patients over 5 months after baseline-testing, we were able to exclude patients who were given a dementia diagnosis during their hospitalization or follow up period.

Our neurocognitive test battery was comprehensive and broad-based, especially in the executive domain. Instead of using just one or two well-known “executive tests,” we created a composite measure consistent with the recommendation in Miyake et al. (2000b). Our patient population was comparable to other relevant study populations with regard to education, gender, estimated IQ and medical morbidity.

There are a number of potential limitations. The control group is small and cognitive impairment is not the defined 10% across domains. It varies from 0 to 18%. On the two domains with evident differences between groups, the percentage of impairment in the HC group was less than 6%. An additional limitation is that the size of the patient group is also rather small. The Low *N* in the patient group may cause reduced power to detect real differences. By reporting effect sizes of group differences, we invite the reader to decide whether non-significant findings represent possible effects given larger subject samples. The mean age of the controls was 3 years older than the patients' and may have exaggerated group differences that were age dependent. This threat to external validity was addressed by using age as a covariate variable in group comparisons.

Results from neurocognitive testing could possibly be influenced by the effect of neurotropic medication. This is difficult to avoid when studying a group of in-patients with mainly long-standing symptoms of a depressive episode. The domain of Language showed a rather weak internal consistency. Each test in this domain should perhaps be separately considered in the later analysis of adverse effects of ECT on neurocognition in elderly patients.

### Conflict of interest statement

The authors declare that the research was conducted in the absence of any commercial or financial relationships that could be construed as a potential conflict of interest.
